# Elotuzumab spares dendritic cell integrity and functionality

**DOI:** 10.1007/s00432-021-03572-z

**Published:** 2021-03-02

**Authors:** Sebastian Schlaweck, Leon Strauss, Solveig Daecke, Peter Brossart, Annkristin Heine

**Affiliations:** Medical Clinic III for Oncology, Hematology, Immune-Oncology and Rheumatology, University Hospital Bonn, Venusberg Campus 1, 53127 Bonn, Germany

**Keywords:** Elotuzumab, Multiple myeloma, Dendritic cells, T cell activation

## To the editor

The armamentarium of proteasome inhibitors and IMiDs for Multiple Myeloma (MM) therapy was recently extended to monoclonal antibodies, which now are even investigated or are already approved as therapeutic option in first-line treatment (Mateos et al. 2018). Among these, the CD38 antibody daratumumab as well as the SLAMF7 antibody elotuzumab have shown remarkable results in MM therapy providing new treatment options. The results from the ELOQUENT2 and ELOQUENT3 study, both investigating the impact of adding elotuzumab to a back bone consisting of immunomodulatory drugs (IMiDs) and steroids extended the therapeutic options for patients suffering from relapsed or refractory MM.

Recently, the results from the ELOQUENT3 study have been supported by real-world data, underlining again that neutropenia and pneumonia are serious side effects of this therapeutic regime (Hose et al. 2021).

MM patients have been described to be per se sevenfold more susceptible to develop infections compared to population controls (Girmenia et al. 2019). Thus, detailed knowledge of potential immunosuppressive side effects of anti-Myeloma therapy is mandatory. Regarding elotuzumab, data about susceptibility for infections is inconsistent. The ELOQUENT3 study did not report any increased risk for infections for patients receiving elotuzumab together with pomalidomide and dexamethasone (Dimopoulos et al. 2018). In contrast, the final results from the ELOQUENT2 trial acknowledged higher infection rates for the experimental group receiving elotuzumab in combination with lenalidomide and dexamethasone (ERd) compared to patients treated with lenalidomide and dexamethasone(Rd) alone (Dimopoulos et al. 2020). In detail, elotuzumab-treated patients were prone to infections in general (84% in ERd vs 75% in Rd) and to pneumonia (22% in ERd vs. 16% in Rd) as well as Herpes zoster reactivation (7% in ERd vs. 2% in Rd) (Dimopoulos et al. 2020). Data elucidating whether the elimination of benign antibody-producing plasma cells, drug-induced neutropenia or additional off target effects contribute to this observation is scarce. However, it is already known that targeting SLAMF7, which is overexpressed on the surface of malignant plasma cells (Hsi et al. 2008), has not only direct effects on myeloma cells but also activates NK cells (Campbell et al. 2018), indicating that elotuzumab does not solely target MM cells, but also other immune cells. Elotuzumab triggers the antibody-dependent cellular cytotoxicity by NK cells and macrophages. Moreover, it promotes co-stimulatory pathways in NK cells myeloma-dependently and –independently by binding SLAMF7 on the NK cell surface (Campbell et al. 2018).

Dendritic cells (DCs) are essential in the induction of appropriate immune responses as they link the adaptive and innate immune system (Carbone et al. 1998), but, commonly used anti-myeloma drugs interfere with DCs. In detail, proteasome inhibitors and IMiDs suppress DC functionality (Nencioni et al. 2006a, b; Yamamoto et al. 2019) as well as daratumumab depletes plasmacytoid DCs and modulates PD-L1 expression on antigen presenting cells (Stocker et al. 2020), while it upregulates CD80 and CD86 on monocytes (Viola et al. 2020). Given the increased infection rate observed in MM patients, we wondered whether elotuzumab modulates DC phenotype, function and their ability to induce T cell responses as well.

**Fig. 1:**
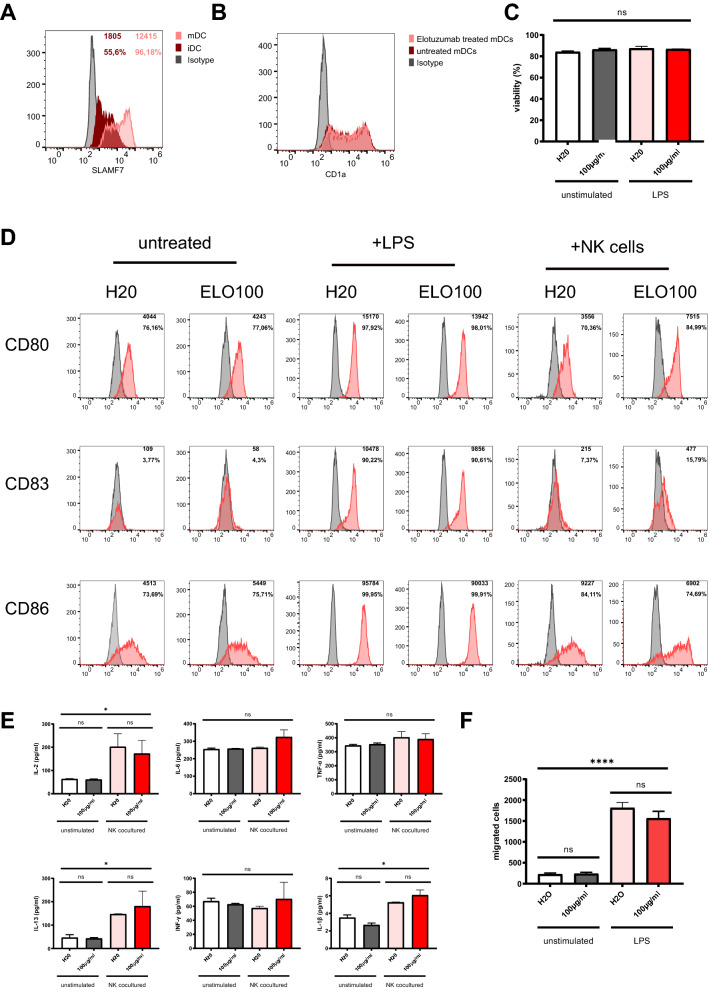
Elotuzumab spares the function of SLAMF7-positive dendritic cells. Human dendritic cells were generated in the presence of IL-4 and GM-CSF from peripheral blood mononuclear cells (PBMCs) harvested from buffy coats of healthy volunteer donors as described previously (Heine et al. 2013). Elotuzumab (100 µg/ml) was added every other day. LPS (100 ng/ml) was added on day 6 to mature iDCs, when indicated. NK cells were harvested from the same buffy coat by magnetic cell separation (NK cell isolation kit, Miltenyi Biotec, Bergisch Gladbach) and frozen until they were utilized. NK—DC cocultures (1:1 ratio) were performed with cells from the same buffy coat. DCs were harvested and analyzed after seven days of culture. All experiments were repeated at least three times with different donors. Representative donors are shown. **a** SLAMF7 expression was measured on immature (dark red) and mature DCs (light red). MFI and percentage expression of immature (dark red) and mature DCs (light red) are depicted. iDCs express SLAMF7, which is upregulated in mDCs (55.6% up to 96.18%). **b** CD1a expression of mature DC either treated with the vehicle H_2_O (dark red) or elotuzumab (100 µg/ml) (light red) is unchanged, indicating that elotuzumab does not interfere with DC differentiation. **c** Viability of DCs generated from PBMCs in the presence of elotuzumab was measured via FACS. A viability above 80% shows no toxic effects of elotuzumab. **d** Expression of CD80, CD83 and CD86 is not inhibited by elotuzumab treatment. Elotuzumab-exposed NK cells induce expression of CD80 (70.36–84.99%) an CD83 (7.37–15.79%), but CD86 expression is slightly dampened (84.11–74.69%). **e** Secretion of pro-inflammatory cytokines is not altered by elotuzumab pre-treatment. NK cells stimulate the secretion of IL-2, IL-1β or IL-13 by DCs, but elotuzumab does not potentiate this effect. **f** CCR7-dependent migration of DCs is not affected by elotuzumab in a transwell assay (8 µm) towards a CCL19 gradient

To investigate the effects of elotuzumab, we isolated peripheral blood mononuclear cells (PBMCs) from buffy coats of healthy volunteers. DCs were generated from PBMCs in the presence of IL-4 and GM-CSF as previously described (Heine et al. 2013). First, we were able to show that immature DCs (iDCs) express relevant SLAMF7 on their surface (Fig. 1a). Toll-like receptor 4 (TLR4) stimulation and subsequent maturation even upregulated SLAMF7 expression [55.6% on iDCs to 96.18% on mature DCs (mDCs)]. Next, we demonstrated that in vitro generation of DCs, measured by expression of the lineage marker CD1a, (Fig. 1b) and viability of human iDCs (Fig. 1c) is not affected by co-incubation with elotuzumab with concentrations up to 150 µg/ml.

When elotuzumab was added during the differentiation period of naïve PBMC to DCs every other day (day 0, 2, 4 and 6) and iDCs were maturated by addition of lipopolysaccharide (LPS) on day 6, the expression of the co-stimulatory molecules CD40, CD80, CD83 and CD86 remained unaffected. iDCs co-incubated with NK cells alone or in the presence of elotuzumab for additional 24 h even showed a slight upregulation of CD80 (70.36–84.99%) and CD83 (7.37–15.79%. Expression of CD40 remained unaffected (99%; data not shown), while CD86 (84.11–74.69%) showed a slight downregulation (Fig. 1d). This indicates that activation markers of DCs are not significantly altered during Elotuzumab treatment.

It has already been shown in detail that binding of elotuzumab to SLAMF7 activates NK cells and that this enhances their interaction with macrophages (Campbell et al. 2018). Data regarding induction of cytokine production by binding of SLAMF7 on DCs or enhanced interaction of NK cells and DC by elotuzumab is lacking. Therefore, iDCs generated as described above were exposed to the SLAMF7 antibody elotuzumab alone or in the presence of NK cells. Interestingly, the presence of NK cells increased the secretion of IL-2, IL-13 and IL-1β. Addition of elotuzumab had no effect on the respective cytokine release. Moreover, NK cells, elotuzumab or a combination of both did not affect cytokine levels of INF-γ, IL-6 and TNFα.

Besides upregulation of activation markers and adequate cytokine production, migration of DC to lymphatic organs is a prerequisite for a robust immune response. Therefore, we investigated the migratory ability of mDCs, generated in the presence or absence of elotuzumab, in a transwell approach against a CCL19 gradient. Here, we could show that the CCR7-CCL19-dependent migration of DCs remains unaffected by elotuzumab (Fig. 1f).

Taken together, we could rule out direct suppressive effects of elotuzumab as well as indirect effects of elotuzumab-pre-treated NK cells on DC phenotype and function, such as cytokine production, chemokine-driven migration, induction of T cell responses and cytotoxicity.

Elotuzumab maintained the functionality of DCs, which is a prerequisite for robust immune responses to infectious pathogens as well as in the context of prophylactic vaccinations. However, the induction and duration of vaccination responses in MM patients has been described to be frequently decreased (Mustafa et al. 2019).Thus, the approach to prevent serious infectious complications by the application of prophylactic vaccinations may not be corrupted by SLAMF7 antibodies, although prior treatments might of course diminish immune responses. Our data shows that DC function is preserved, thus implicating that vaccination may be a feasible approach during elotuzumab treatment. Moreover, elotuzumab seems to be safe regarding promising novel therapeutic approaches for the treatment of MM containing vaccination regimes (Perumal et al. 2020).

In summary, we add insight into the immune shaping effects of MM treatment. Elotuzumab spares dendritic cell functionality and integrity but has previously been shown to stimulate NK cell function. Hence, elotuzumab might be a feasible option in MM patients at special risk for infections or the need for vaccination.

## Data Availability

Data is available upon request to the corresponding author.
